# Targeted Delivery of STING Agonist via Albumin Nanoreactor Boosts Immunotherapeutic Efficacy against Aggressive Cancers

**DOI:** 10.3390/pharmaceutics16091216

**Published:** 2024-09-17

**Authors:** Zhijun Miao, Xue Song, Anan Xu, Chang Yao, Peng Li, Yanan Li, Tao Yang, Gang Shen

**Affiliations:** 1 Department of Urology, The Fourth Affiliated Hospital of Soochow University, Suzhou 215000, China; 13771818504@163.com (Z.M.); 13956280997@163.com (C.Y.); jmarlipeng@163.com (P.L.); 2 Jiangsu Key Laboratory of Neuropsychiatric Diseases, College of Pharmaceutical Sciences, Soochow University, Suzhou 215123, China; xsong@suda.edu.cn (X.S.); 20214226024@stu.suda.edu.cn (A.X.); ynli@suda.edu.cn (Y.L.); 3 State Key Laboratory of Radiation Medicine and Protection, Soochow University, Suzhou 215123, China

**Keywords:** human serum albumin, targeted delivery, STING agonist, immunotherapy

## Abstract

**Background:** Activating the cytosolic innate immune sensor, the cGAS-STING pathway, holds great promise for enhancing antitumor immunity, particularly in combination with immune checkpoint inhibitors (ICIs). However, the clinical application of STING agonists is often hindered by poor tumor accumulation, limited cellular uptake, and rapid clearance. To address these challenges, we developed a human serum albumin (HSA)-based nanoreactor system for the efficient delivery of the STING agonist SR-717, aiming to improve its antitumor efficacy. **Methods:** Using a biomineralization technique, we encapsulated SR-717 within HSA nanocages to form SH-NPs. These nanoparticles were characterized in terms of size, stability, and cellular uptake, and their ability to activate the STING pathway was assessed in both in vitro and in vivo models, including freshly isolated human renal tumor tissues. In vivo antitumor efficacy was evaluated in a murine renal tumor model, and immune responses were measured. **Results:** SH-NPs exhibited enhanced stability, efficient cellular uptake, and superior tumor accumulation compared to free SR-717. They robustly activated the STING pathway, as evidenced by increased phosphorylation of TBK1 and IRF3, along with elevated IFN-β production. Additionally, SH-NPs reshaped the immunosuppressive tumor microenvironment, promoting T-cell-mediated immunity and improving the therapeutic efficacy of checkpoint blockade in murine models. The validation in human renal tumor tissues further highlighted their potential for clinical translation. Importantly, SH-NPs were well tolerated with minimal systemic toxicity. **Conclusions:** This study underscores the potential of HSA-based nanoparticles for the targeted delivery of STING agonists, effectively enhancing antitumor immunity and improving cancer immunotherapy outcomes. SH-NPs offer a promising solution to the limitations of current STING agonists in clinical settings.

## 1. Introduction

Immunotherapy, emerging as a prominent strategy in cancer treatment, has garnered significant attention and research efforts [1,2,3]. Novel immune treatments for oncology, such as mAbs, aiming at CTLA-4 and the PD-1/PD-L1 axis, exemplify the advances in innovative cancer immunotherapies [4,5,6] and have revolutionized the landscape of cancer therapy. Immune checkpoint inhibitors (ICIs) circumvent the inhibitory checkpoint molecules on tumor cells, thereby enhancing T-cell responsiveness and preventing immune exhaustion, thereby restoring immune surveillance [7,8,9]. Despite achieving clinical success, these therapies demonstrate considerable variability in their response rates across patients [10,11]. For instance, the response rate to ipilimumab is only about 15%, and is rarely more than 25% in patients receiving anti-PD-1/PD-L1 ICI therapy [12,13,14]. Immune checkpoint inhibitor (ICI) efficacy is inherently linked to the existing pro-inflammatory features of the tumoral microenvironment (TME), often marked by the substantial infiltration of immune cells and increased levels of PD-L1 expression. Conversely, tumors classified as “immune-desert” or “cold” display a significantly reduced response to ICIs, thereby presenting greater challenges for achieving effective treatment [15,16,17]. To fully harness the potential of immune checkpoint inhibitor (ICI) therapy, it is crucial to convert “cold” tumors into a more immunogenic and pro-inflammatory phenotype—termed “hot”. This transformation can revitalize antitumor immunity, thereby improving the therapy’s effectiveness. A particularly viable approach to achieve this involves enhancing the activity of the cGAS-STING pathway. This pathway is a vital component of the innate immune system, playing crucial roles in both antiviral defense and antitumor response [18,19]. Serving as a guardian of the innate immune system, the cGAS-STING pathway excels at detecting cytoplasmic, double-stranded DNA (dsDNA). Upon recognizing such exogenous genetic material, it initiates a series of complex downstream signaling events, eventually leading to a robust immune response aimed at combating infections [20]. This pathway bridges innate and adaptive immunity, facilitating the activation and migration of DCs and the subsequent initiation of CTLs at tumor sites [21]. Thus, this pathway has the potential to overcome immune-suppressive environments in certain cancers, rendering patients more responsive to immune checkpoint inhibitor (ICI) therapy [22]. Triggering the response of the innate immune system through exogenous stimulation with STING agonists, such as cyclic GMP-AMPP, has proven effective in enhancing the efficacy of immunotherapy [23]. However, STING agonists based on cyclic dinucleotides (CDNs) are small molecules with hydrophilic negative charges, making them prone to enzymatic degradation and limiting their activation of the STING pathway in target tissues [24,25]. Non-nucleotide STING agonists like SR-717 have shown potential in overcoming the instability associated with cyclic dinucleotide (CDN)-based agonists [26]. Nevertheless, these drugs are hydrophobic, which contributes to their poor targeting and side effects, restricting their clinical utility [27]. Therefore, there is a critical need to develop new treatment strategies that are capable of improving drug solubility and targeting specificity and reduce the side effects.

A targeted delivery system has been widely utilized in biomedical research to address these challenges [28,29,30]. Many researchers have proposed various drug delivery strategies aimed at improving STING agonists’ utilization efficiency to enhance the antitumor immune effects. For example, Zhou et al. used PMOF nanoparticles for light-triggered STING agonist SR-717 release, enhancing photodynamic-immune therapy [31]. Lu et al. engineered hollow manganese dioxide nanoparticles that incorporate the STING agonist MSA-2 and the CRISPR-Cas9/sg-PD-L1 plasmid for prolonged tumor release. This formulation activates the cGAS-STING pathway and downregulates PD-L1 expression [32]. Additionally, a biomimetic cancer-cell-membrane-coated nanovaccine delivery system (PLGA/STING@EPBM) effectively delivered STING agonists and tumor antigens to Clec9a^+^ DCs, showing significant antitumor synergy with radiotherapy [33]. These advancements in nanocarrier development effectively address the limitations of STING agonists in clinical applications caused by delivery barriers and adverse reactions. However, the complex preparation process and challenges in translation to clinical practice remain significant drawbacks.

**Scheme 1:**
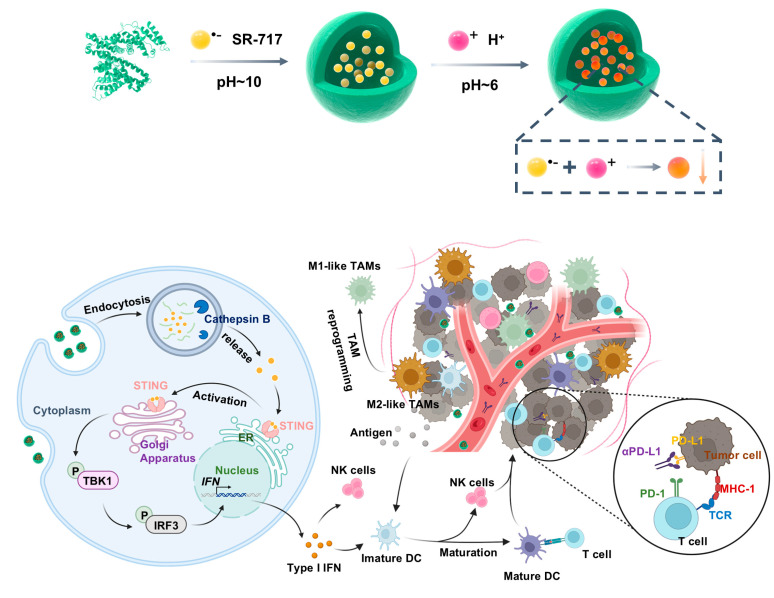
The synthesis process of using human serum albumin (HSA) as a single−molecule nanoreactor to encapsulate the STING agonist SR−717, and the subsequent process of immune activation in tumor immunotherapy.

In recent years, our research has focused on developing innovative protein-based nanomedicine delivery systems with excellent targeting capabilities, a high drug loading efficiency, and improved safety, significantly enhancing the efficacy of tumor therapy [34,35]. Building on this foundation, we have also explored the use of human serum albumin as a nanoreactor at the single-molecule level. By leveraging biomineralization principles, we have achieved the controlled nucleation and crystallization of therapeutic agents within the protein nanocage, resulting in multifunctional protein nanoparticles with tunable dimensions. These nanoparticles hold significant promise for targeted cancer therapies, including chemotherapy, photothermal therapy, and photodynamic therapy [36,37,38]. Herein, we employed a biomineralization approach using a single-molecule albumin template to encapsulate the STING agonist SR-717 (SH-NPs). These nanoparticles exhibited excellent tumor targeting ability with superior serum stability and efficient cellular uptake, thus considerably activating the STING signaling pathway, followed by relieving the immunosuppression and improving the immunogenetic feature of TME. Finally, SH-NPs enhanced T-cell mediated antitumor immunity and augmented the effectiveness of checkpoint blockade therapy. Their efficacy was confirmed in freshly isolated human renal tumor tissues, underscoring their potential for clinical application. These findings pave the way towards targeted STING agonist delivery for the reinvigorated immunotherapy of intractable cancers (Scheme 1).

## 2. Materials and Methods

### 2.1. Materials

The SR-717 was obtained from Selleck Co., Ltd (Houston, TX, USA). The human serum albumin (HSA) was sourced from Aladdin Reagent (Shanghai, China). The RPMI 1640 medium, fetal bovine serum (FBS), trypsin-EDTA solution, and penicillin-streptomycin were from Gibco (Billings, MT, USA). The IL-4 and IFN-γ proteins were from Peprotech (Rocky Hill, NJ, USA). The Cy5.5 NHS ester and LPS proteins were from Sigma (St. Louis, MO, USA). The ELISA kits for IFN-β, CXCL-10, IL-6, IL-1β, and TNF-α were sourced from Lianke (Shanghai, China). The antibodies for Phospho-TBK1 (5483S), Phospho-IRF3 (29047S), TBK1 (38066S), and IRF3 (4550S) from CST (Danvers, MA, USA) and for GAPDH (AC033) from Abclonal (Wuhan, China) were used. The goat anti-rabbit IgG H&L secondary antibody (ab6702) was obtained from Abcam (Cambridge, UK). Additionally, various monoclonal antibodies from BioLegend (San Diego, CA, USA) were used: PE anti-mouse CD80 (104708), APC anti-mouse CD86 (105012), PE anti-mouse CD86 (105007), PE anti-mouse CD206 (141706), FITC anti-mouse CD45 (103108), APC anti-mouse CD3 (100236), PE anti-mouse CD8a (100708), PE anti-mouse CD335 (137604), FITC anti-mouse CD11c (117306), FITC anti-mouse/human CD11b (101206), APC anti-mouse F4/80 (123116), APC-Cy7 anti-mouse CD4 (100414), PE anti-mouse Foxp3 (126404), PE anti-mouse CD11b (101207), APC anti-mouse Gr-1 (108412), FITC anti-human CD14 (982502), APC anti-human CD68 (333809), PE anti-human CD86 (374205), and PE anti-human CD206 (321105).

### 2.2. Cell Lines

The RENCA (CL-0568), DC2.4 (SCC142), and RAW264.7 (K1673) cell lines from the Shanghai Cell Bank were cultured in RPMI 1640 with 10% FBS at 37 °C, 5% CO_2_.

### 2.3. Animals and Ethics Statement 

The BALB/c mice (aged 6–8 weeks, 18 ± 2 g, female) from Shanghai SLAC were housed at 21 ± 1 °C, 40–70% humidity, on a 12 h light–dark cycle, with ad libitum food and water. All the procedures were conducted per the approved protocols from Soochow University’s Laboratory Animal Center (ECSU-2019000179). The maximum allowable tumor volume was set at 1500 mm^3^, adhering to ethical guidelines to ensure the welfare of the mice.

### 2.4. Synthesis 

To synthesize SH-NPs, a 1.0 mL liquid of SR-717 at 1.0 mg/mL concentration was added to a mixture including 30.0 mg of HSA in 3.0 mL of DI water under stirring, and the pH was set to 10. After the HSA fully dissolved, the pH was reduced to 6. The mixture was exposed to treatment at 55 °C for 4 h. Post treatment, the SH-NPs were separated using centrifugation and ultrafiltration, with a membrane cutoff of 100 kDa at a g-force of 1000 g, for 20 min per round, repeated five times. The SH-NPs, after being purified, were stored in a PBS solution at pH 7.4 with a molarity of 10.0 mM.

### 2.5. Characterization

The shape characteristics of the SH-NPs were analyzed by transmission electron microscopy (TEM) (HT7700, Hitachi, Tokyo, Japan). Concurrently, the particle size and surface charge were measured by dynamic light scattering (DLS) (Zetasizer ZS90, Malvern, Worcestershire, UK). The level of SR-717 was quantified by the use of RP-HPLC, using the Agilent 1100.

### 2.6. Drug Release

Using the dialysis method, the release behavior of SR-717 in free SR-717 and SH-NPs was studied. A volume of 1.0 mL of the specimen, containing 0.2 mg/mL of SR-717, was enclosed in dialysis bags with a MWCO of 3.5 kDa. These drug-loaded bags were subsequently submerged in a pH 7.4 phosphate buffer, a pH 5.0 acetate buffer, and a pH 5.0 acetate buffer with 10 μg/mL of CB and then shaken with an oscillator at 37 °C. The level of SR-717 in the buffer was determined at 0, 0.5, 1, 2, 4, 8, 12, and 24 h.

### 2.7. Cellular Uptakes and Endocytic Pathway

To examine SH-NP internalization, the DC2.4 cells (1.0 × 10^6^ per well) were seeded and treated with either free SR-717 or SH-NPs (both 10.0 µM SR-717), then incubated for 12 or 24 h. Post incubation, the cells were lysed via ultrasonication, and the SR-717 was quantified using HPLC. For endocytic mechanism studies, the cells were pretreated with chlorpromazine (10.0 µg/mL), amiloride (100.0 µg/mL), or nystatin (5.0 µg/mL) for 1 h at 37 °C or 4 °C. After the inhibitor pretreatment, cells were incubated with SH-NPs (10.0 µM SR-717) for 12 h, lysed, and analyzed by HPLC to determine the uptake pathways.

### 2.8. In Vitro Cytotoxicity

To investigate the cytotoxic effects, the DC2.4 cells were initially plated at a density of 1.0 × 10^4^ cells per well. Subsequently, the cells were incubated with free SR-717 and SH-NPs, each at concentrations of 0, 1.25, 2.5, 5.0, 10.0 and 20.0 µM SR-717 for 24 h. The cell viability was assessed using the MTT colorimetric assay.

### 2.9. Western Blot

The DC2.4 cells were incubated with free SR-717 and SH-NPs (10.0 µM SR-717) for 24 h. Total proteins were isolated from the cells utilizing a Protein Extraction Kit provided by Beyotime (Shanghai, China). The protein concentrations were quantified using the BCA assay. For the analysis, 20 µg of protein per sample was separated on a 10% SDS-PAGE gel and transferred to PVDF membranes. The membranes were blocked with 5% BSA for 2 h at room temperature, then incubated overnight at 4 °C with primary antibodies (anti-Phospho-TBK1, anti-Phospho-IRF3, anti-TBK1, anti-IRF3, and anti-GAPDH). After washing with TBST, membranes were incubated for 1 h at room temperature with HRP-conjugated goat anti-rabbit IgG secondary antibodies. Following additional washes, the protein bands were visualized using an ECL detection system. For the protein extraction from tumor tissues, female BALB/c mice with RENCA tumors received 30 mg/kg SH-NPs. The tumors were excised, disrupted in CHAPS solution, centrifuged, and stored at −80 °C.

### 2.10. DC Maturation

For the DC stimulation experiments, the DC2.4 cells were incubated with free SR-717 and SH-NPs (10.0 µM SR-717) for 24 h. Then the DCs were stained with anti-CD86 PE and anti-CD80 APC and analyzed by a flow cytometer to investigate their maturation via the surface expression of the costimulatory molecules CD86 and CD80. Additionally, the levels of IFN-β in the culture medium were measured by ELISA kits (Lianke, Shanghai, China).

### 2.11. Macrophage Polarization

For the macrophage polarization experiments, M1 macrophages were induced with 100.0 ng mL^−1^ of LPS and 20.0 ng mL^−1^ of IFN-γ, and M2 macrophages were induced with 20.0 ng mL^−1^ of IL-4. Polarized macrophage phenotypes were identified using flow cytometry. Subsequently, the M2 macrophages were incubated with free SR-717 and SH-NPs (10.0 µM SR-717) for 24 h. Following incubation, the RAW264.7 cells were stained with anti-CD206 PE and anti-CD86 PE and analyzed by a flow cytometer to investigate their polarization via the surface expression of the costimulatory molecules CD206 and CD86.

### 2.12. The Construction of RENCA Tumor Models 

To create the subcutaneous tumor models, BALB/c mice (aged 6–8 weeks, female) were inoculated with 5.0 × 10^6^ RENCA tumor cells via subcutaneous injection at the right dorsal region.

### 2.13. In Vivo Tumor Targeting 

To evaluate the tumor targeting efficacy in RENCA tumors, BALB/c mice (aged 6–8 weeks, female) with RENCA tumors were intravenously injected with Cy5-SH-NPs at a dosage of 30.0 mg/kg SR-717. Then fluorescence images were taken at intervals of 0, 2, 6, 12, 24, and 48 h and analyzed using Living Imaging software, version 4.4.

### 2.14. In Vivo Biodistribution

To investigate the biodistribution in RENCA tumors, BALB/c mice (aged 6–8 weeks, female) with RENCA tumors were intravenously injected with either free SR-717 or SH-NPs at a dosage of 30.0 mg/kg SR-717. Each group consisted of three mice. At 12 h post-injection, SR-717 was extracted from the heart, liver, spleen, lung, kidney, and tumor tissues using methanol and quantified by HPLC.

### 2.15. In Vivo Antitumor Efficacy

For evaluating the antitumor efficacy, mice with RENCA tumors were treated with either free SR-717 or SH-NPs at a dose of 30.0 mg/kg. Each treatment group included five mice. Following intravenous administration, the tumor volume was regularly monitored and calculated using the appropriate formula V = 0.5 × L × W^2^, where V denotes the tumor volume, L is the length, and W is the width.

For the investigation of the combined antitumor effects of SH-NPs and aPD-L1 in subcutaneous RENCA tumor models, the mice with tumors ranging from 75 to 100 mm^3^ received intravenous administrations of SH-NPs (30.0 mg/kg SR-717) and aPD-L1 (3.0 mg/kg). Twenty-four hours post initial treatment, a supplementary dose of aPD-L1 (3.0 mg/kg) was given intravenously. The tumor volumes were also quantified using the same formula as described above, with each experimental cohort having five mice.

### 2.16. Cytokine Analysis

To evaluate the cytokine analysis, free SR-717, SH-NPs (30.0 mg/kg SR-717), and aPD-L1 (3.0 mg/kg) were administered intravenously to the mice bearing RENCA tumors. Each experimental group consisted of three mice. Twenty-four hours post initial treatment, a supplementary dose of aPD-L1 (3.0 mg/kg) was given intravenously. The mice were euthanized, and blood was collected at 3 days post treatment, after which plasma was obtained by centrifugation. Then, the levels of IFN-β, CXCL-10, IL-6, and TNF-α were measured by ELISA kits (Lianke, Shanghai, China).

### 2.17. Antitumor Immune Response

In summary, the mice with RENCA tumors (*n* = 3 per cohort) received intravenous injections of free SR-717 and SH-NPs containing 30 mg/kg SR-717. Following the initial treatment, an intravenous dose of 3.0 mg/kg aPD-L1 was administered 24 h later. To evaluate the anti-cancer immune reaction in vivo, lymph nodes and tumors were removed from the BALB/c mice (aged 6–8 weeks, female) with subcutaneous RENCA tumors three days post treatment. The specimens were prepared for individual cell suspensions by mechanical or enzymatic dissociation in staining buffers. To analyze the immune cell populations in the tumors, cells were labeled with specific fluorescent antibodies for 60 min and examined via flow cytometry. The CTLs and NK cells were labeled with FITC-CD45, APC-CD3, PE-CD8a, and PE-CD335 antibodies. The lymph node DCs were stained with FITC-CD11c, PE-CD80, and APC-CD86. The TAMs were labeled with FITC-CD11b, APC-F4/80, PE-CD206, and PE-CD86. The CD4^+^ T-cells and Tregs were labeled with FITC-CD45, APC-CD3, APC-Cy7-CD4, and PE-Foxp3. The MDSCs were labeled with FITC-CD45, PE-CD11b, and APC-Gr-1. A flow cytometry was performed using FACS Aria III, and the data were analyzed with FlowJo^TM^ v10 software.

### 2.18. The Evaluation of Antitumor Immune Response in Resected Human Tissues

Informed consent was obtained from patients for the utilization of their biospecimens in this research, a protocol that was sanctioned by the Fourth Affiliated Hospital of Soochow University. Human tissues, specifically renal cell carcinoma (RCC), were excised and immediately cleansed before being dissected into smaller segments (ranging from 1 to 5 mm^3^) with a surgical blade. Within half an hour post-resection, these tissue pieces were treated with either SR-717 or SH-NPs encapsulating 500 µg of SR-717 and diluted in a 5% glucose solution. The tissue fragments were incubated in an RPMI 1640 medium with supplements for 24 h. The tissues were digested with collagenase IV and DNase I then filtered for single-cell suspension. The cells were labeled with specific antibodies and analyzed by flow cytometry. Finally, the IFN-β levels in the medium were measured using ELISA kits from Lianke.

### 2.19. Safety Studies

To assess liver function, the mice were humanely euthanized 24 h post-administration, and blood samples were carefully collected. The serum was isolated via centrifugation, and the concentrations of alanine aminotransferase (ALT) and aspartate aminotransferase (AST) were measured using an automated biochemical analyzer. To evaluate inflammation in the liver and spleen, the mice were euthanized either 24 h or 21 days post-administration. The liver and spleen tissues were homogenized to create tissue suspensions. The supernatant from these homogenates was separated by centrifugation, and the cytokines IL-1β and IL-6 were quantitatively analyzed using an ELISA kit.

### 2.20. Statistics and Reproducibility

The data were analyzed using GraphPad Prism 8.0.2. In vitro and in vivo experiments were repeated at least three times for validity and reliability. The results are shown as mean ± SD. The sample sizes (*n*) denote biological duplicates, determined from exploratory results. The subjects were randomly assigned to experimental groups without blinding. All the data were included in the analysis. The statistical significance between two groups was evaluated using an unpaired, two-tailed Student’s *t*-test. For multiple groups, a one-way ANOVA followed by a Tukey’s post hoc test were used. The survival rates were assessed using Kaplan–Meier and log-rank tests. The statistical significance was set at *p* < 0.05.

## 3. Results

### 3.1. The Preparation and Characterization of SH-NPs

**Figure 1:**
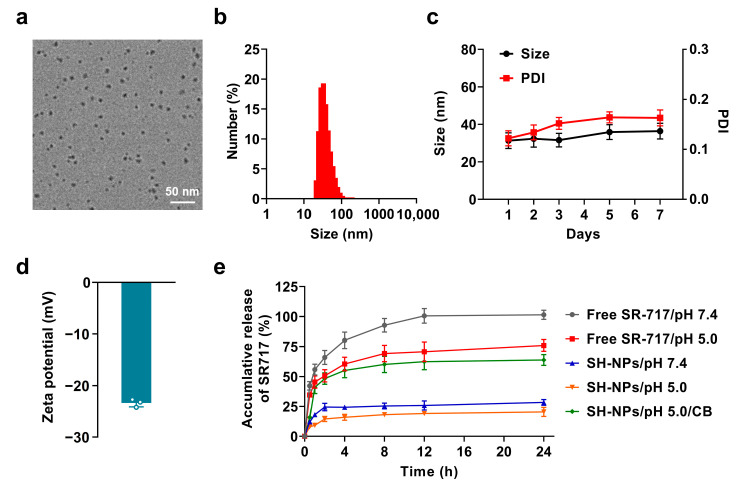
Characterization of SH−NPs. TEM image (**a**) and hydrodynamic size distribution (**b**) of SH−NPs; (**c**) hydrodynamic size of SH−NPs stored at 4 °C for 7 days; (**d**) eta potential of SH−NPs; (**e**) accumulative release of SR−717 from free SR−717 and SH−NPs in pH 7.4 phosphate buffer, pH 5.0 acetate buffer, and pH 5.0 acetate buffer containing 10 μg mL^−1^ of CB.

In a typical synthesis process, human serum albumin was applied as a single-molecule nanoreactor to encapsulate SR-717. First, SR-717 was dissolved in basic solutions and incubated with albumin under stirring, followed by adjusting the pH to 6 to allow the quick precipitation of SR-717 inside the albumin nanocages. The obtained SH-NPs were then purified via ultracentrifuge and stored for further use. As shown in Figure 1a, the SH-NPs showed a round-like morphology with the size of 5.6 ± 1.8 nm. Furthermore, the result from the dynamic laser scanning revealed that the diameter of SH-NPs was calculated to be 31.3 ± 4.2 nm with the PDI value of 0.122 (Figure 1b), indicating a uniform size distribution. The SH-NPs exhibited satisfactory stability in aqueous solutions (Figure 1c), as evidenced by the negligible variations in their hydrodynamic size over the course of 7 days when stored at 4 °C. Also, the surface potential of the SH-NPs was negative, with the value of −23.4 ± 0.7 mV (Figure 1d), implying that the negative surface charge facilitates the formation of stable complexes with positively charged serum proteins, potentially extending the circulation time and enhancing the nanoparticles’ ability to reach target tissues [39]. Afterwards, we further applied the dialysis method to investigate the drug release behavior of SH-NPs in the presence and absence of cathepsin B (CB). We found that the accumulative release amount of SR-717 from the SH-NPs remained relatively low during 24 h in a pH 7.4 solution or a pH 5.0 solution (Figure 1e). By contrast, free SR-717 was rapidly released, with the amount reaching 90%. Furthermore, the release behavior of the SH-NPs was accelerated in the presence of cathepsin B (Figure 1e), which resulted from the responsive degradation of the albumin.

### 3.2. STING Activation and Immune Activation of SH-NPs inside Immune Cells

**Figure 2:**
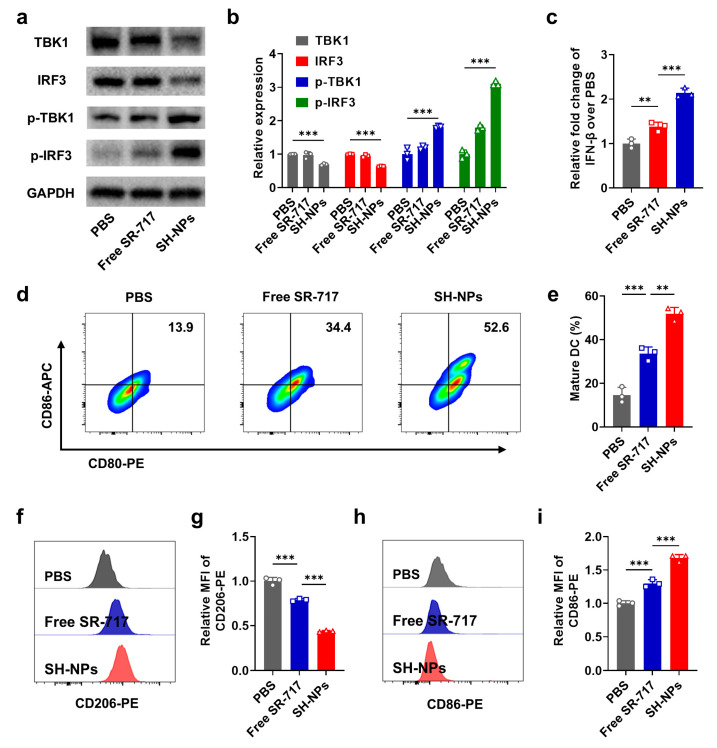
Cellular behaviors of SH−NPs. Expression of downstream proteins of cGAS−STING signaling pathway including p−TBK1 and p−IRF3 (**a**) and statistics of protein relative gray value (**b**); (**c**) concentration of IFN−β secreted by cells treated with free SR−717 and SH−NPs; the expression of CD80 and CD86 on the surface of DC2.4 after administration (**d**) and statistics of maturation ratio (**e**); (**f**−**i**) expression and the fluorescence intensity of CD206 (**f**,**g**) and CD86 (**h**,**i**) on the surface of macrophages after incubation with free SR−717 and SH−NPs. Statistical differences: ** *p* < 0.01, *** *p* < 0.001 (Figure 2b,c,e,g,i).

To unravel the STING activation efficiency of the SH-NPs, we first evaluated their cellular uptake in DC cells. As exhibited in Supplementary Figure S1, the SH-NPs showed time-dependent internalization into immune cells during 24 h incubation, and the cellular uptake amount was significantly higher than that of free SR-717, which is ascribed to the clathrin-mediated endocytosis (Supplementary Figure S2). We then tested the cytotoxicity of the SH-NPs and no obvious cell death was found during 24 h, even at the high concentration of 20 μM (Supplementary Figure S3), indicating their superior safety and biocompatibility. We proceeded to examine the phosphorylation state of TBK1 and IRF3, both essential for the function of the STING pathway. After a 24 h treatment with the SH-NPs at a concentration of 10 μM, a significant rise in the amounts of p-TBK1 and p-IRF3 was detected. Notably, the p-TBK1 and p-IRF3 levels were elevated by 1.5-fold and 1.7-fold, respectively, relative to the levels triggered by free SR-717 (Figure 2a,b). Since the p-TBK1 and p-IRF3 triggered the subsequent production of IFN-β proteins, we conducted an enzyme-linked immunosorbent assay (ELISA) to measure IFN-β release. The SH-NPs, due to the increased cell uptake and the higher levels of p-TBK1 and p-IRF3, caused a significant rise in IFN-β production. Specifically, the IFN-β levels were elevated by 2.2-fold relative to the untreated group and by 1.4-fold compared to the free SR-717 group (Figure 2c).

Next, we sought to investigate the immune activation of the SH-NPs using the DCs and the macrophages as model cells. Prior to the effective activation of the STING pathway, the SH-NPs remarkably increased the critical co-stimulation signals (CD80 and CD86) in the cell surface, indicating the maturation of the DC cells for subsequent antigen presentation (Figure 2d). However, the DC cells that were incubated with free SR-717 failed to induce any maturation, owing to their weak IFN-β production (Figure 2e). Additionally, the membrane expression of CD206, a recognized indicator for M2-polarized macrophages, showed a notable reduction in macrophages after the SH-NPs treatment (Figure 2f,g). Conversely, the expression of CD86, a key indicator for M1-polarized macrophages, increased by 1.3-fold compared to the levels seen in the free SR-717 treatment group (Figure 2h,i). This significant change indicates a possible repolarization or attraction of the macrophages, thus diminishing their immunosuppressive function. These data indicated that the STING activation of the SH-NPs would potently stimulate an immunogenic tumor immune microenvironment with relieved immunosuppression, which is highly important for improving the cancer immunotherapeutic efficacy.

### 3.3. Tumor Accumulation and the Antitumor Efficacy of SH-NPs

**Figure 3:**
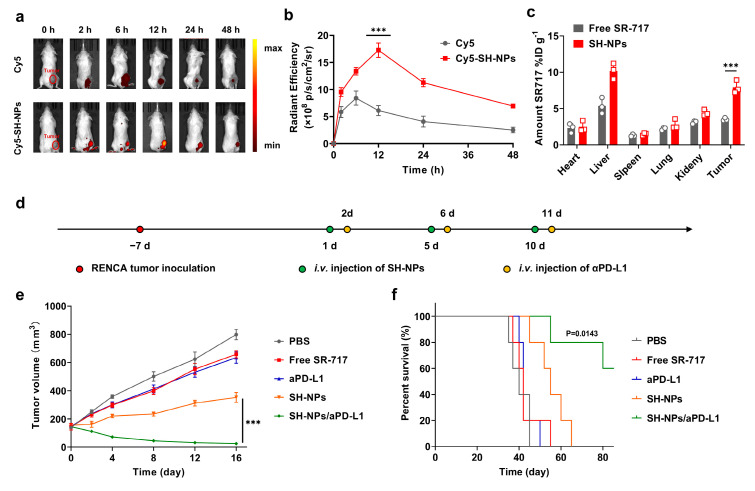
Tumor targeting and in vivo antitumor efficacy of SH−NPs. In vivo fluorescence imaging of renal tumor bearing mice receiving SH−NPs at different time points (**a**) and the fluorescence intensity of tumor region (**b**); (**c**) tumor accumulation amount of SR−717 in the renal tumor bearing mice at 12 h post−injection of SH−NPs; (**d**) timeline schedule of treatment of renal tumor bearing mice; (**e**,**f**) tumor growth profiles (**e**) and survival curve (**f**) of the mice bearing renal tumor treated with PBS, free SR−717, aPD−L1, SH−NPs and SH−NPs/aPD−L1. Statistical differences: *** *p* < 0.001 (Figure 3b,c,e).

To understand the in vivo performance of the SH-NPs, we created the subcutaneous-renal-tumor-bearing mice to evaluate the tumor targeting capability of the SH-NPs. We first covalently conjugated a fluorescent dye (Cy5) to the albumin shell of the SH-NPs for visualizing their in vivo biodistribution. As described in Figure 3a, the SH-NPs gradually accumulated in the tumor region and reach their peak at 12 h post-injection, with 2.8 times the fluorescence intensity of free Cy5, suggesting their superior targeting ability for maximizing the antitumor efficacy. Furthermore, we employed HPLC to quantitatively analyze the distribution of the SH-NPs in tumors and major organs, including the heart, liver, spleen, lungs, and kidneys. In consistence with the previous result, the SH-NPs showed excellent tumor accumulation, with up to 7.9 ID%/g, being a 2.2-fold increase compared to free SR-717 (Figure 3c). Due to a significant drug accumulation in the liver at 12 h, further investigation was conducted on the levels of the liver injury markers ALT and AST in the serum of the mice after administration. The results indicated that the free SR-717 and the SH NPs treatment groups did not exhibit significant increases in ALT and AST (Supplementary Figure S4). Simultaneously, the mouse livers were homogenized to measure the levels of the inflammatory factors IL-1β and IL-6. The results, in Supplementary Figure S5, indicated that only the free SR-717 treatment group exhibited a slight increase in IL-1β, while the IL-6 levels were significantly elevated. These results suggest that the free SR-717 and SH NPs treatment groups exhibited no significant potential toxicity or off-target effects and demonstrated good biocompatibility.

Inspired by the excellent tumor-targeting capability identified, we proceeded to assess the antitumor efficacy of the SH-NPs in the mice with renal tumors. When the tumor volume reached 140–150 mm^3^, the SH-NPs were administered intravenously at a dose of 30.0 mg/kg. The tumor volumes were then monitored over a period of 16 days (Figure 3d). As shown in Figure 3e,f, the renal tumors receiving the treatment of free SR-717 exhibited an aggressive growth profile, and all the mice died with 55 days. In contrast, the SH-NPs significantly delayed the tumor growth, probably owing to their enhanced tumor targeting and subsequent STING pathway activation (Figure 3e). Considering the elevated PD-L1 expression after STING activation, we further applied an anti-PD-L1 antibody for synergistic therapy. The combination of the anti-PD-L1 and the SH-NPs did not result in complete tumor eradication. Nonetheless, it is noteworthy that this synergistic treatment regimen conferred a significant survival benefit, with 60% of the mice surviving past 80 days. In contrast, monotherapy with either the SH-NPs or the anti-PD-L1 exerted only a limited therapeutic effect on the renal tumor growth. (Figure 3e). These data imply that the SH-NPs with remarkable tumor targeting would induce a potent antitumor effect and could further improve the therapeutic efficacy of a checkpoint blockade against murine renal cancers.

### 3.4. STING Activation and Immune Responses of the SH-NPs in Mice Bearing a Renal Tumor

**Figure 4:**
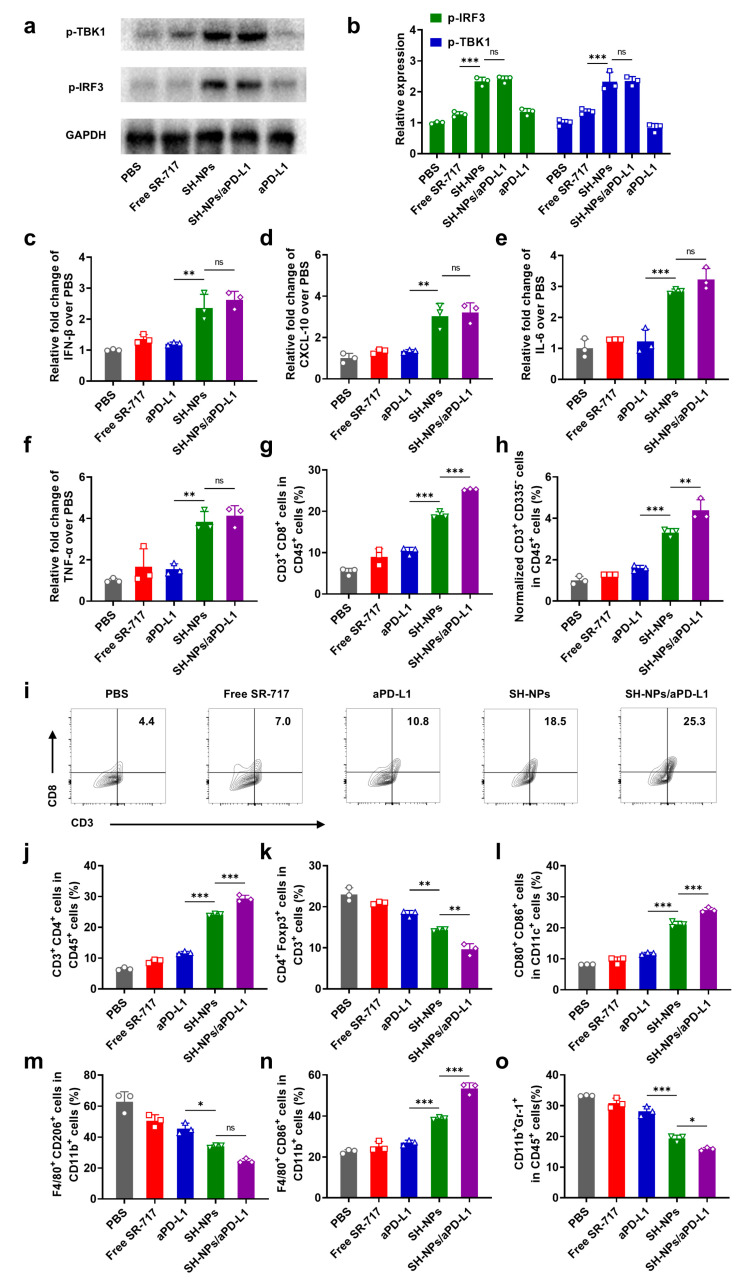
In vivo STING activation and immune responses. (**a**,**b**) Expression (**a**) and the relative protein gray value (**b**) of p−TBK1 and p−IRF3 in tumor tissue from the mice bearing renal tumors that were treated with PBS, free SR−717, aPD−L1, SH−NPs, and SH−NPs/aPD−L1 at 72 h post−injection; (**c**−**f**) concentration of IFN−β (**c**), CXCL−10 (**d**), IL−6 (**e**), TNF−α (**f**) in tumor tissue from the same set of mice, assessed at the same time point; (**g**−**i**) quantification of tumor−infiltrating CTLs (**g**,**i**) and NK cells (**h**) in tumor tissue; (**j**,**k**) quantification of T−cells (**j**) and Tregs (**k**) in tumor tissue; (**i**) quantification of matured dendritic cells inside tumor−draining lymph nodes; (**m**,**n**) quantification of M2−like TAMs (**m**) and M1−like TAMs (**n**) in tumor tissue; (**o**) quantification of MDSCs in tumor tissue. Statistical differences: * *p* < 0.05, ** *p* < 0.01, *** *p* < 0.001 (Figure 4b−h,j−o), ns-not significant (Figure 4b–e).

To unravel the detailed mechanism of the antitumor efficacy in the mice receiving the SH-NPs treatment, we carefully assessed the STING activation and immune responses after various treatments. First, the essential proteins, including p-TBK1 and p-IRF3, were evaluated via western blotting. In the mice treated with free SR-717 or anti-PD-L1, p-TBK1 and p-IRF3 were slightly upregulated, while the SH-NPs significantly elevated their expressions with 1.7-fold and 1.8-fold increases as compared to that of the free SR-717 groups (Figure 4a), which was ascribed to their enhanced tumor targeting and cellular uptake. As expected, a much higher secretion of IFN-β was observed in the mice receiving the SH-NPs treatment, regardless of its combination with anti-PD-L1 or not. Meanwhile, chemoattractants such as CXCL10 (Figure 4d), closely associated with T-cell proliferation and function, along with cytokines including IL-6 (Figure 4e) and TNF-α (Figure 4f), were elevated post treatment with the SH-NPs, a result of the robust activation of the STING pathway. Then, we collected the tumors from the mice treated with various formulations and analyzed the population of various immune cells at 3 days after injection. First, the SH-NPs induced higher tumor-infiltrating cytotoxic T-cells marked by CD45^+^CD3^+^CD8^+^ T-cells, being a 2.1-fold and 1.8-fold increase as compared to those of free SR-717 and anti-PD-L1 (Figure 4g,i), respectively. Meanwhile, another injection of anti-PD-L1 further elevated the infiltration of the T-cells, probably due to the immune checkpoint blockade. As a typical phenotype of an innate immune system, natural killer (NK) cells showed a similar trend, in which free SR-717 or anti-PD-L1 failed to arouse any significant improvement, while the highest infiltration amount of NK cells was observed in the mice treated with the SH-NPs/anti-PD-L1. In particular, the NK cell proportion increased by a substantial 3.4-fold relative to the free SR-717 treatment and by 2.7-fold compared to the anti-PD-L1 treatment alone, underscoring the synergistic impact of the combined therapeutic approach (Figure 4h and Figure S6a,c). The SH-NPs/anti-PD-L1 treatment group increased the proportion of CD4^+^ T-cells in mouse tumors to approximately 30% (Figure 4j and Figure S7a,c) and significantly reduced the proportion of regulatory T-cells (Tregs) (Figure 4k and Figure S7b,d). Furthermore, we also picked out the lymph nodes and tested the maturation of the DCs after STING activation. Obviously, a 2.2-fold higher DCs maturation was observed in the mice receiving the SH-NPs as compared to those receiving free SR-717 (Figure 4i and Figure S6b,d), suggesting the targeted delivery of a STING agonist is highly advantageous for immune activation. Notably, our findings indicate that combining SH-NPs with anti-PD-L1 significantly enhances the maturation of DCs. This effect is likely attributable to the intrinsic expression of PD-L1 on the surface of DCs [40].

Given that the immunosuppressive microenvironment impedes immunotherapy, we evaluated tumor-associated macrophages (TAMs) with different phenotypes: CD11b^+^F4/80^+^CD206^+^ (M2-like, immunosuppressive) and CD11b^+^F4/80^+^CD86^+^ (M1-like, pro-inflammatory). The SH-NPs (spherical nanoparticles) treatment significantly increased M1-like TAMs and decreased M2-like TAMs. The ratio of CD11b^+^F4/80^+^CD86^+^ to CD11b^+^F4/80^+^CD206^+^ TAMs showed a 6.0-fold increase in the SH-NPs compared to the controls (Figure 4m,n and Figure S8). This shift towards an antitumor phenotype suggests that SH-NPs enhance immunotherapy effectiveness. The SH-NPs and anti-PD-L1 synergistic treatment group significantly reduced the proportion of MDSCs, outperforming the single SH-NPs treatment group, and mitigated the immunosuppressive tumor microenvironment (Figure 4o and Figure S9).

To evaluate the long-term toxicity and immunogenicity of free drugs and nanoparticles, they were administered via the tail vein in the mice on days 1, 3, and 5. The secretion of inflammatory factors and the enrichment of immune cells in the liver and spleen were assessed on day 21. As shown in Supplementary Figure S10a,b, the free drug induced the secretion of IL-1β and IL-6 in the liver at 21 days, with an increase of 1.3 and 2.0 times compared to the PBS group, and the same was observed in the spleen. There was no significant increase in the SH-NPs treatment group. Free SR-717 also led to the increased infiltration of immune cells in organs, whereas the SH-NPs did not. The results confirmed that the SH-NPs did not cause long-term toxicity or increased immunogenicity in organs, demonstrating the potential of albumin nanocarriers in significantly improving the biosafety of the drug.

### 3.5. STING Activation in Human Tissues

**Figure 5:**
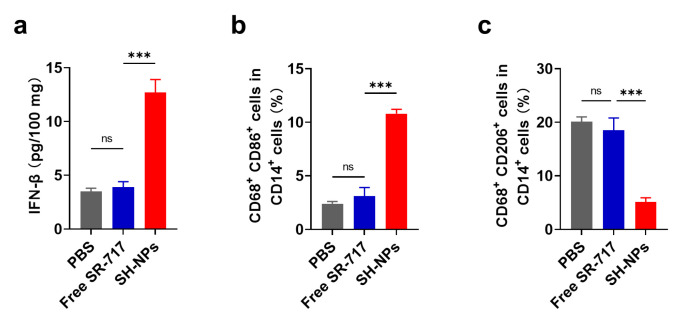
STING activation in freshly isolated human renal tumor tissues. (**a**−**c**) Concentration of IFN−β CD14b^+^CD68^+^CD86^+^ TAMs (**b**) and CD14b^+^CD68^+^CD206^+^ TAMs (**c**) in human renal tumor tissues treated with intratumoral injection of PBS, free SR−717, and SH−NPs. Statistical differences: *** *p* < 0.001, ns-not significant (Figure 5a−c).

To investigate the translational potential, we examined the feasibility of activating the STING pathway in human tissues. Freshly excised primary renal cell carcinoma (RCC) tissues were used in our study. These tissues received local injections of either free SR-717 or SH-NPs, followed by an assessment of IFN-β secretion. Due to limited bioavailability, free SR-717 had a negligible effect on IFN-β expression compared to the control. In stark contrast, the SH-NPs induced a significant increase in IFN-β production, with a 3.6-fold increase relative to the control and a 3.2-fold enhancement over the free SR-717 group (Figure 5a). Notably, the markers indicative of M1 macrophages showed a significant rise, while the markers for M2 macrophages declined significantly following the SH-NPs treatment compared to other groups. (Figure 5b,c). This suggests that the SH-NPs effectively activated STING in the human RCC tissues, thereby significantly enhancing IFN-β secretion and modulating macrophage polarization.

## 4. Discussion

We have developed HSA-based nanoparticles as a highly effective approach to deliver the STING agonist SR-717, which significantly improved the cellular uptake, stability, and STING activation of SR-717, leading to enhanced antitumor immune responses both in vitro and in vivo. These findings support the potential of SH-NPs as a promising delivery system for cancer immunotherapy. Previous studies have highlighted the challenges associated with the delivery of STING agonists, particularly regarding their stability and targeting specificity. Traditional STING agonists, such as CDNs, suffer from rapid degradation and limited efficacy due to poor tissue penetration and off-target effects [24,25]. Non-nucleotide STING agonists like SR-717 have shown potential but still face issues related to hydrophobicity and targeting [26,41]. Our approach using HSA-based nanoparticles addresses these challenges by enhancing the solubility and targeting efficiency of SR-717. Our research revealed that SH-NPs not only improve the stability and cellular uptake of SR-717 but also significantly activate the STING pathway, as evidenced by the elevated levels of phosphorylated TBK1 and IRF3 (Figure 2a,b). This activation leads to the production of IFN-β proteins (Figure 2c), crucial for initiating antitumor immune responses. The enhanced STING activation further promotes the maturation of DCs and the polarization of macrophages towards a pro-inflammatory phenotype (Figure 2d,i). These immunological changes contribute to a more immunogenic tumor microenvironment, facilitating the recruitment and activation of CTLs and NK cells, which are essential for effective tumor eradication (Figure 4g,h). The in vivo studies in murine models demonstrated that the SH-NPs significantly enhanced tumor targeting and retention of SR-717, resulting in a prolonged circulation time and a higher tumor accumulation compared to free SR-717 (Figure 3a). This targeted delivery translated into superior antitumor efficacy, with the SH-NPs effectively delaying tumor growth and improving survival rates in renal-tumor-bearing mice. The combination of the SH-NPs with anti-PD-L1 antibodies further potentiated the antitumor effects, leading to significant tumor regression in some cases (Figure 3f). These results underscore the potential of SH-NPs in synergistic cancer immunotherapy.

In conclusion, this research underscores the potential of HSA-based nanoparticles in delivering STING agonists specifically to targeted sites, offering a promising method to boost the effectiveness of cancer immunotherapy. Our results open new avenues for creating more effective and precise cancer treatments, potentially revolutionizing the field.

## Data Availability

The data that support the findings of this study are available from the corresponding author upon reasonable request.

## Supplementary Materials

The following supporting information can be downloaded at https://www.mdpi.com/article/10.3390/pharmaceutics16091216/s1. Figure S1: Amount of internalized SR-717 of free SR-717 or SH-NPs by DC2.4 cells at different time points; Figure S2: Amount of internalized SR-717 of DC2.4 cells incubated with SH-NPs and various endocytic pathway inhibitors; Figure S3: Cell viability of DC2.4 cells treated with free SR-717 or SH-NPs; Figure S4: Expression of ALT and AST in serum as biomarkers of liver damage in mice bearing renal tumors 24 h post-injection of either free SR-717 or SH-NPs; Figure S5: IL-1β and IL-6 expressed in the liver in mice bearing renal tumors 24 h post-injection of either free SR-717 or SH-NPs; Figure S6: Representative flow cytometric plots and percentages of CD45^+^CD3^−^CD335^+^ NK cells (**a**,**c**) in RCC tumors and matured dendritic cells (CD11c^+^CD80^+^CD86^+^ DCs) (**b**,**d**) inside tumor-draining lymph nodes; Figure S7: Representative flow cytometric plots and percentages of CD45^+^CD3^+^CD4^+^ T-cells (**a**,**c**) and CD3^+^CD4^+^Foxp3^+^ Tregs (**b**,**d**) inside RCC tumors; Figure S8: Representative flow cytometric plots and percentages of CD11b^+^F4/80^+^CD206^+^ TAMs (**a**,**c**) and CD11b^+^F4/80^+^CD86^+^ TAMs (**b**,**d**) inside RCC tumors; Figure S9: Representative flow cytometric plots and percentages of CD45^+^CD11b^+^Gr-1^+^ MDSCs (**a**,**b**) inside RCC tumors; Figure S10: IL-1β and IL-6 expressed in the liver (**a**) and spleen (**b**) at 21 days following the administration of three doses and(**c**) the immune cell infiltration ratio in the liver and spleen at 21 days following the administration of three doses.
