# Supplementary material for: Targeted Delivery of STING Agonist via Albumin Nanoreactor Boosts Immunotherapeutic Efficacy against Aggressive Cancers
Source: Pharmaceutics. 2024 Sep 17;16(9):1216. doi: 10.3390/pharmaceutics16091216 (PMC11434985; doi:10.3390/pharmaceutics16091216)
Supplement: Supplementary file 1 [file pharmaceutics-16-01216-s001.zip › pharmaceutics-3120722-supplementary.pdf]

pharmaceutics

Supplementary Materials: Targeted Delivery of STING Agonist via Albumin Nanoreactor Boosts Immunotherapeutic Efficacy against Aggressive Cancers

Zhijun Miao, Xue Song, Anan Xu, Chang Yao, Peng Li, Tao Yang, and Gang Shen

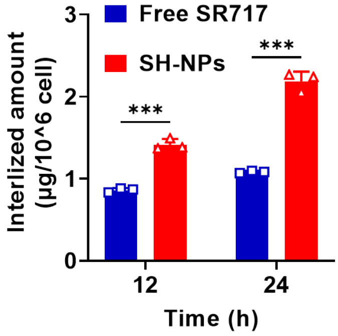

Figure S1. Amount of internalized SR717 of free SR-717 or SH-NPs by DC2.4 cells at different time points. Statistical differences: *** $p < 0.001$

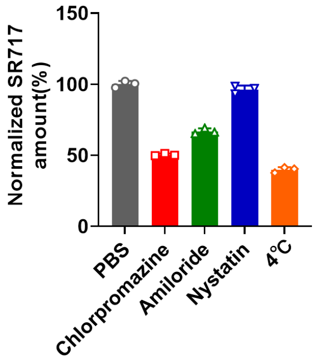

Figure S2. Amount of internalized SR-717 of DC2.4 cells incubated with SH-NPs and various endocytic pathway inhibitors.

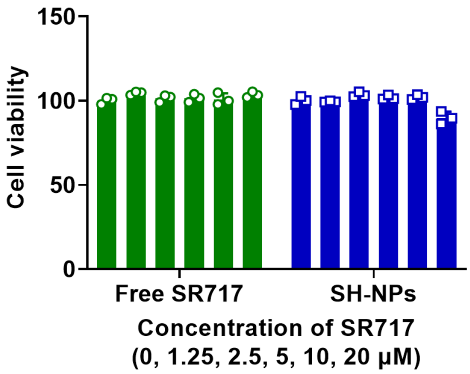

Figure S3. Cell viability of DC2.4 cells treated with free SR-717 or SH-NPs.

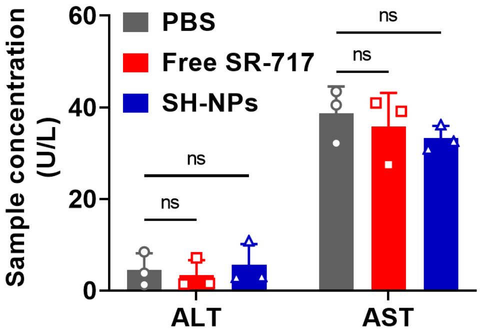

Figure S4. Expression of ALT and AST in serum as biomarkers of liver damage in mice bearing renal tumors 24 h post-injection of either free SR-717 or SH-NPs. Statistical differences: ns-not significant.

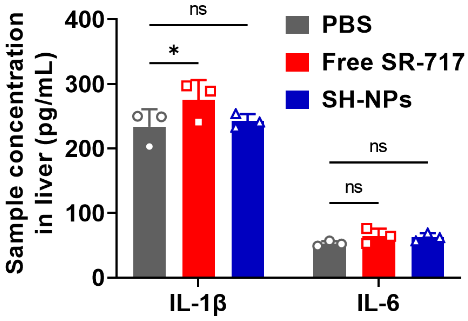

Figure S5. IL-1β and IL-6 expressed in the liver in mice bearing renal tumors 24 h post-injection of either free SR-717 or SH-NPs. Statistical differences: * $p < 0.05$, ns-not significant

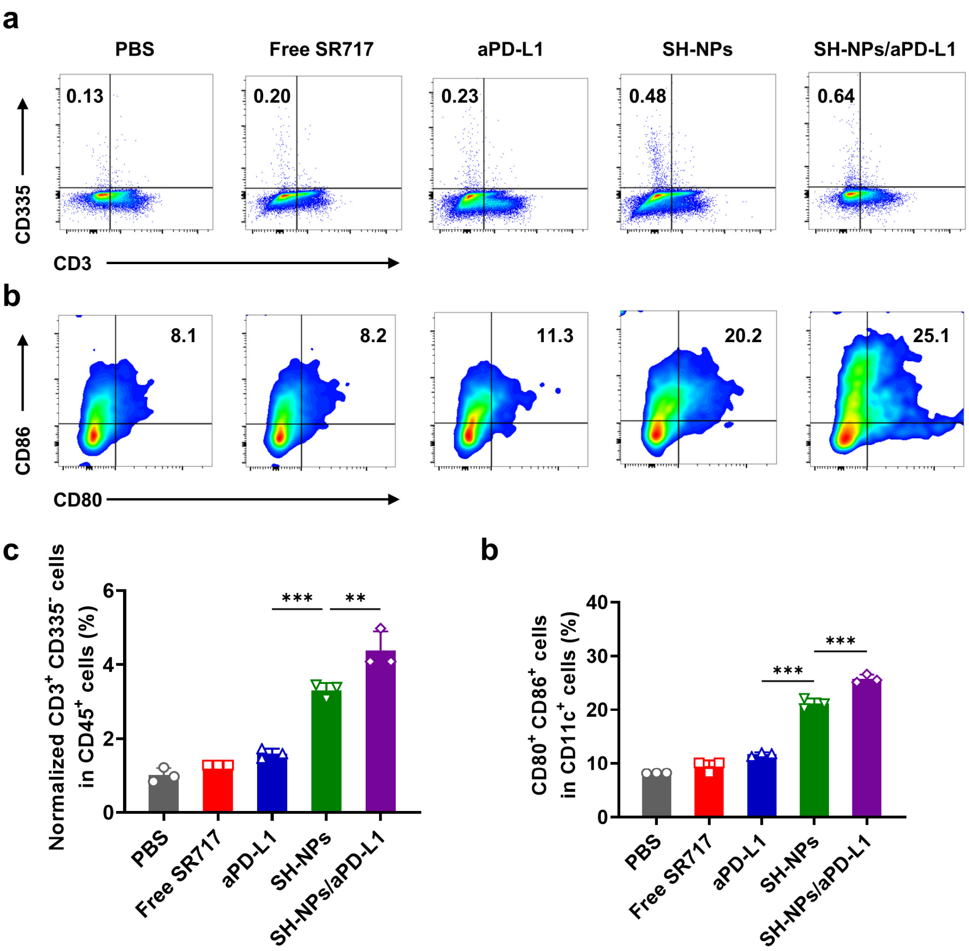

Figure S6. Representative flow cytometric plots and percentages of CD45$^+$CD3$^-$CD335$^+$ NK cells (a, c) in RCC tumors and matured dendritic cells (CD11c$^+$CD80$^+$CD86$^+$ DCs) (b, d) inside tumor-draining lymph nodes. Statistical differences: ** $p < 0.01$, *** $p < 0.001$.

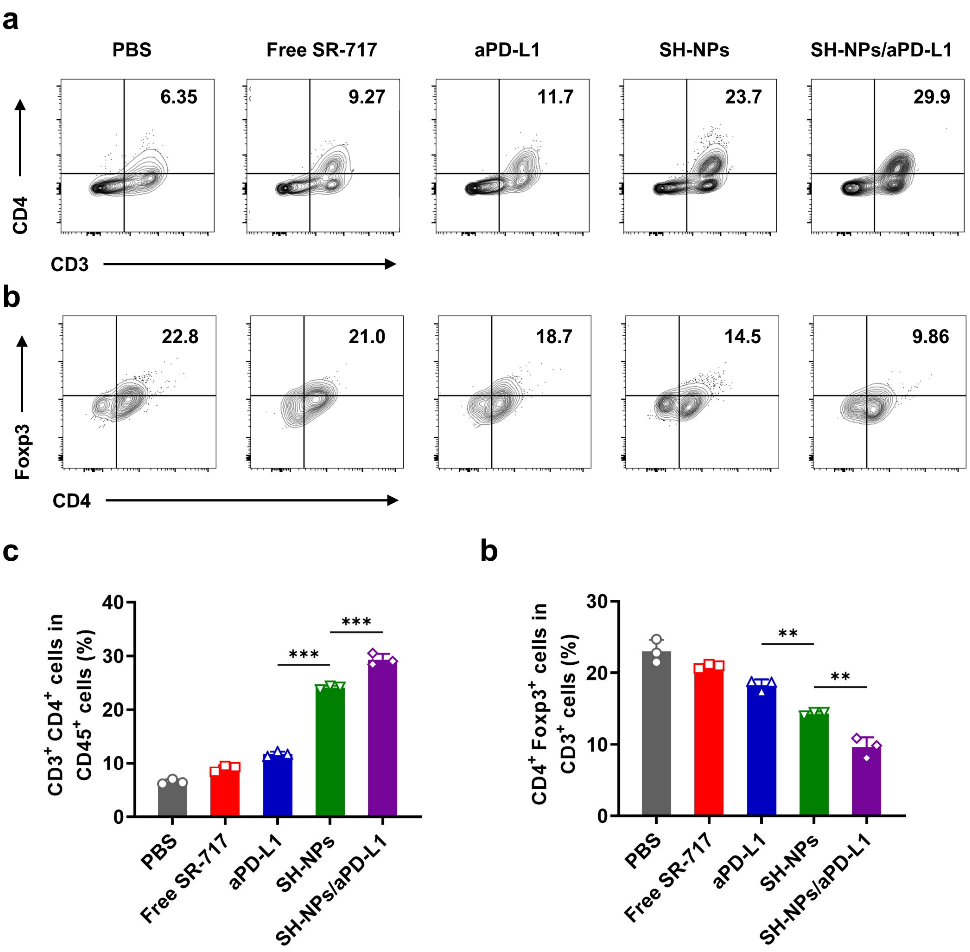

Figure S7. Representative flow cytometric plots and percentages of CD45⁺CD3⁺CD4⁺ T cells (a, c) and CD3⁺CD4⁺Foxp3⁺ Tregs (b, d) inside RCC tumors. Statistical differences: ** $p < 0.01$, *** $p < 0.001$.

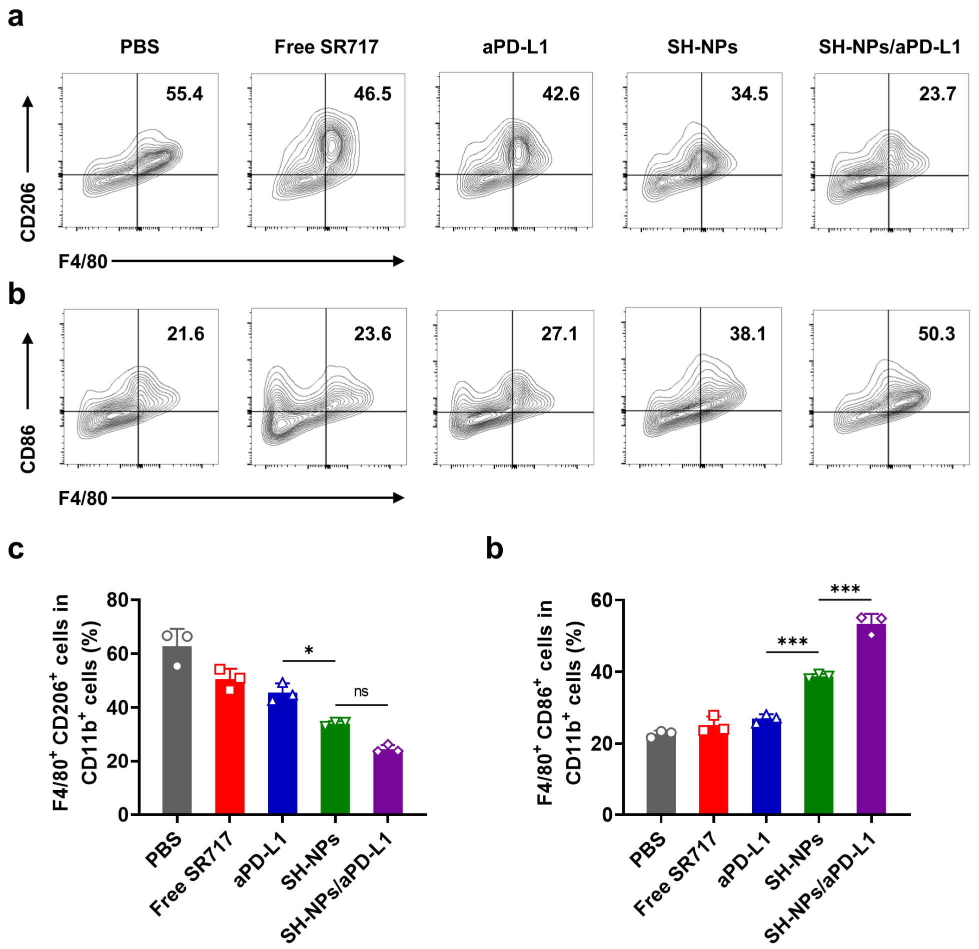

Figure S8. Representative flow cytometric plots and percentages of $CD11b^+F4/80^+CD206^+$ TAMs (a, c) and $CD11b^+F4/80^+CD86^+$ TAMs (b, d) inside RCC tumors. Statistical differences: * $p < 0.05$, *** $p < 0.001$, ns-not significant.

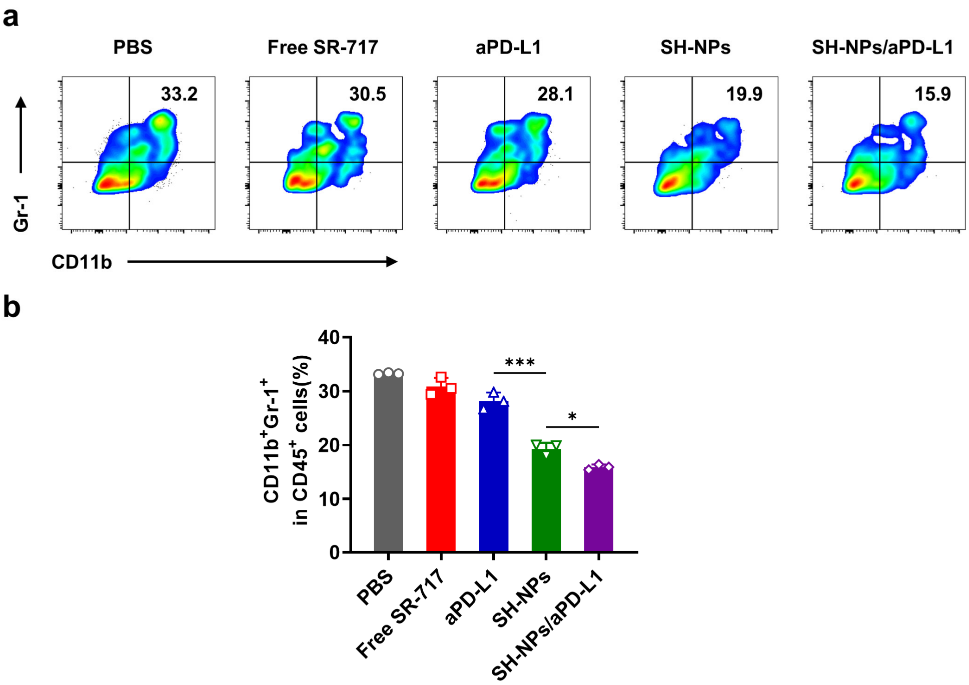

Figure S9. Representative flow cytometric plots and percentages of CD45⁺CD11b⁺Gr-1⁺ MDSCs (a, b) inside RCC tumors. Statistical differences: * $p < 0.05$, *** $p < 0.001$.

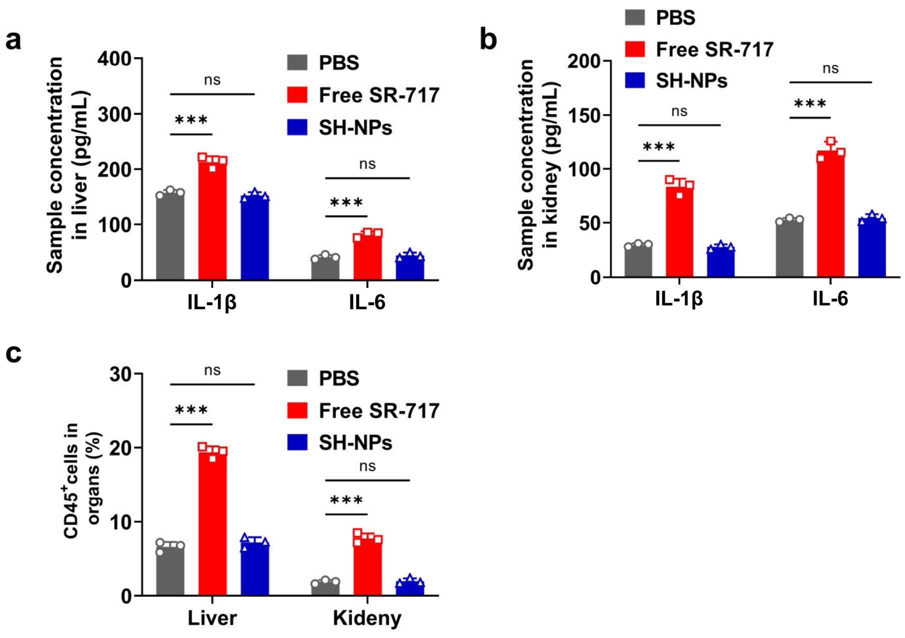

Figure S10. IL-1β and IL-6 expressed in the liver (a) and spleen (b) at 21 days following the administration of three doses; (c) The immune cell infiltration ratio in the liver and spleen at 21 days following the administration of three doses. Statistical differences: *** $p < 0.001$, ns-not significant.
